# CoQ10-loaded liposomes combined with UTMD prevented early nephropathy of diabetic rats

**DOI:** 10.18632/oncotarget.24363

**Published:** 2018-01-19

**Authors:** Pian-Pian Chen, He-Lin Xu, Ting-Yue, De-Li ZhuGe, Bing-Hui Jin, Qun-Yan Zhu, Bi-Xin Shen, Li-Fen Wang, Cui-Tao Lu, Ying-Zheng Zhao, Xiao-Kun Li

**Affiliations:** ^1^ Department of Pharmaceutics, School of Pharmaceutical Sciences, Wenzhou Medical University, Wenzhou City, Zhejiang Province, China; ^2^ The First Affiliated Hospital, Wenzhou Medical University, Wenzhou City, Zhejiang Province, China

**Keywords:** diabetic nephropathy, ultrasound-mediated microbubble destruction, coenzyme Q10, color doppler, preventive treatment

## Abstract

Nephropathy is one of the most severe complications of diabetic patients. The therapeutic strategies for diabetic patients should not only focus on the control of blood glucose but also pay attention to the occurrence of diabetic nephropathy (DN). Coenzyme Q10 (CoQ10) has great therapeutic potential for DN. However, the clinical application of CoQ10 has been limited because of its low water-solubility and non-specific distribution. Liposomes were supposed to be an effective way for delivering CoQ10 to kidney. CoQ10 was effectively encapsulated into the liposome (CoQ10-LIP) with a high entrapment efficiency of 86.15 %. The CoQ10-LIP exhibited a small hydrodynamic diameter (180 ± 2.1 nm) and negative zeta potential (−18.20 mV). Moreover, CoQ10-LIP was combined with ultrasound-mediated microbubble destruction (UTMD) to enhance specific distribution of CoQ10 in kidney. In early stage of diabetic mellitus (DM), rats were administrated with CoQ10-LIP followed by UTMD (CoQ10-LIP+UTMD) to prevent occurrence of DN. Results revealed that CoQ10-LIP+UTMD effectively prevented the renal morphology and function of diabetics rats from damage. The protective mechanism of CoQ10-LIP was highly associated with protecting podocyte, promoting vascular repair and inhibiting cell apoptosis. Conclusively, CoQ10-LIP in combination with UTMD might be a potential strategy to prevent occurrence of DN.

## INTRODUCTION

The incidence of type-1 diabetes mellitus (DM) is increasing worldwide both in low and high income populations [1]. Type-1 DM can afflict people of any age, but usually occurs in children or young adults [2]. Diabetic nephropathy (DN) is one of the most severe complications of DM, which decreases the quality of life and life span of patients, ranking first among other angiopathies. DN is characterized by proliferation of mesangial cells, mesangial hypertrophy and extracellular matrix (ECM) accumulation [3].

It has been found that hyperglycemia is a major cause of increased advanced glycation end products which is responsible for the cause of renal dysfunction in diabetes [4, 5]. Even with the best available management such as tight control of blood pressure and glycemic control, only 30% of opportunity is achieved to improve the declining kidney function as the result of diabetes [6]. Early diagnosis and prevention of DN is key to reduce or delay the progression in diabetic kidney disease [7]. Al-Waili, et al. [8] used natural antioxidants to prevent diabetic nephropathy, and obtained ideal protective effect through regulation of KEAP1/Nrf2/ARE pathway and reduction of inflammatory process. Gu, et al. [9] adopted olmesartan to prevent microalbuminuria in db/db diabetic mice, and also demonstrated olmesartan inhibited the apoptosis of podocyte through inhibition of angiotensin II/p38/SIRT1. Mahfoz, et al. [10] also employed aliskiren to achieve the protective effects on nephropathy of streptozotocin-induced diabetic female rats. Moreover, this research team proved the protective mechanism was highly associated with preserving hemodynamic changes and alleviating oxidative stress. It is urgently required for diabetic patients to prevent occurrence of DN through early intervention of therapeutic agents.

Previous studies have shown that the development of hyperglycemia in DM is followed by the induction of free radical oxidation in the kidneys [11, 12]. Coenzyme Q10 (CoQ10), a vitamin-like antioxidant, present in most eukaryotic cells, primarily in the mitochondria [13], is the only endogenously synthesized antioxidant existing in all cell membranes of our body [14]. As an antioxidant, CoQ10 could control the blood glucose and prevent the occurrence of DN through elimination of free radicals [15]. Some other studies also revealed that CoQ10 had a remarkable curative effect on DN through alleviating glomerulosclerosis, relieving tubular vacuolization, reducing interstitial fibrosis, protecting podocyte and inhibiting cell apoptosis [4, 15, 16]. However, its poor solubility limits the absorption of CoQ10 for oral administration and poor stability in blood circulation system for intravenous injection.

Liposomes, phospholipid bilayer vesicle, have been extensively studied as drug carriers for a few decades, because of its non-toxicity and non-immunogenicity to human body. They can accommodate both hydrophilic and lipophilic candidates, and control release of their encapsulated contents [17]. Encapsulation of CoQ10 by liposomes improved its water solubility and stability *in vivo* and *in vitro*, which was considered to be a promising delivery means [18]. However, CoQ10-loaded liposome was nonspecifically distributed *in vivo* and exhibited a short half-life in blood [19]. It is important to find a novel method to delivery drugs targeted to the kidney and reduce the impact on other body tissues.

Ultrasound (US) consists of pressure waves, which can be focused, reflected and refracted through a medium [20–23]. Thus, US can be carefully controlled and focused on the targeted site or a particular tissue volume within the body. Microbubbles (MBs), which are known as diagnostic ultrasound contrast agents, have been developed to enhance the echogenicity of blood and to delineate the vasculature of tissues [24]. MBs can serve as a vehicle of therapeutics to obtain specific release when exposed to low-intensity ultrasound, resulting in drug accumulation in nidus [25]. Recently, low-intensity ultrasound (US) in combination with microbubbles has been shown to improve the efficiency and tissue/organ specificity of nanoliposomes *in vivo* [26]. Ultrasound-mediated microbubble destruction (UTMD) is a new and promising technique applied in many fields. For example, UTMD has been used to deliver drugs and genes to the cardiovascular system; to overcome blood-brain barrier and blood-tumor barrier; to enhance capillary and vascular permeability enhancement; and to home stem cells [27]. Upon exposure to the low intensity ultrasound, the oscillating microbubbles can impose a destructive stress on the cells to enhance the transport of macromolecular drugs across cellular membrane [28, 29]. Therefore, UTMD holds considerable promise as an effective strategy to achieve targeted delivery of CoQ10-LIP to the kidney.

In the present work, liposomes were used to encapsulate CoQ10 not only to improve its water solubility and stability *in vivo* and *in vitro*, but also enhance its preventive effect on the occurrence of DN. Furthermore, UTMD applied to this system to enhance specific distribution of CoQ10 in kidney. The *in vitro* sustained-release profile of CoQ10-LIP was carefully investigated. The *in vivo* preventive effect of CoQ10-LIP was also thoroughly studied. Finally, the protective mechanism of CoQ10-LIP was also explored.

## RESULTS

### The characterization of CoQ10-LIP

**Figure 1:**
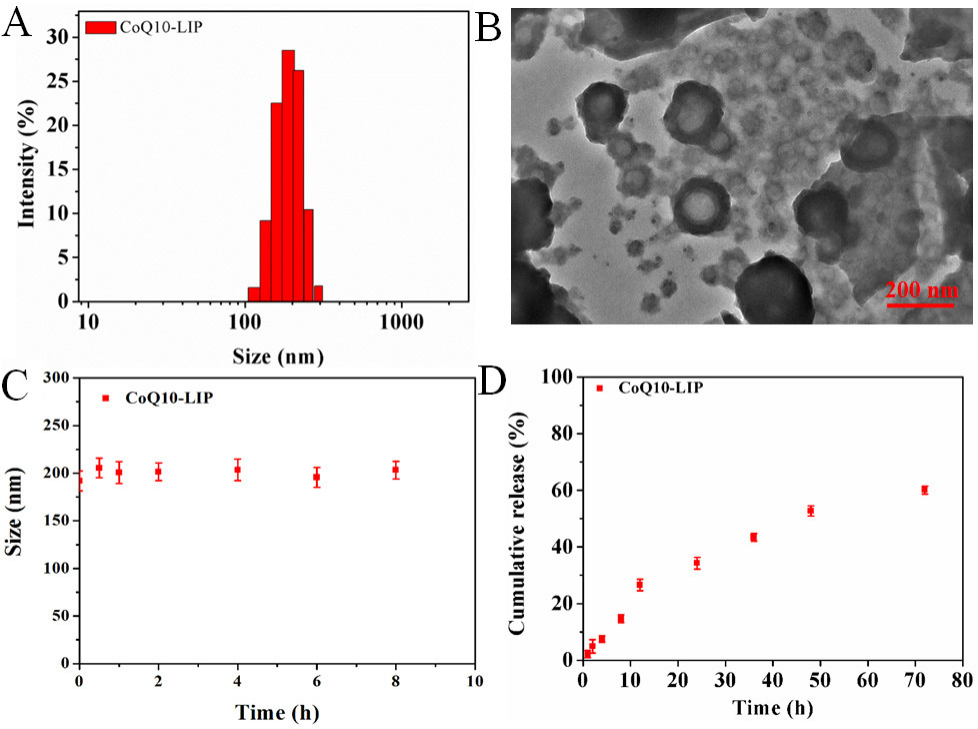
The characterization of CoQ10-LIP (**A**) the particle size distribution, (**B**) TEM graphic, (**C**) the stability of CoQ10-LIP in simulated biological fluid (SBF) containing 5% bovine serum albumin (BSA), and (**D**) the cumulative release profile in pH 7.4 PBS.

The characterization of CoQ10-lip was summarized in Figure 1A. The average particle sizes of CoQ10-lip were 180 ± 2.1 nm, and the particle size distribution index (PDI) was 0.12. The zeta potential of CoQ10-LIP was determined to be −18.20 mV, suggesting its good stability. The encapsulation efficiency of the CoQ10-lip was reaching to 86.15 % (*n* = 3). TEM graphic of CoQ10-LIP was shown in Figure 1B. Most of CoQ10-LIP exhibited almost spherical core-shell morphology. The stability of CoQ10-LIP in simulated biological fluid (SBF) containing 5% bovine serum albumin (BSA) was studied by DLS. As shown in Figure 1C, the particle size and size distribution did not significantly change within 8 h at 37°C, which indicated that the morphological structure of CoQ10-LIP was stable.

### *In vitro* release of CoQ10 from CoQ10-LIP

*In vitro* drug release profiles of CoQ10-LIP in pH 7.4 PBS was shown in Figure 1D. A sustained-release profile of CoQ10 from CoQ10-LIP was observed, without obvious burst release. The cumulative release percentage was only 26.60 ± 2.01 % at 12 h, and even at 72 h only 60.10 ± 1.42% of the encapsulated CoQ10 was released. The sustained-release profile was beneficial to prolong the retention time in blood, which could facilitate more drugs distributing to the damaged kidney.

### Effects of CoQ10-LIP on morphology and function of kidney of diabetics rats

**Figure 2:**
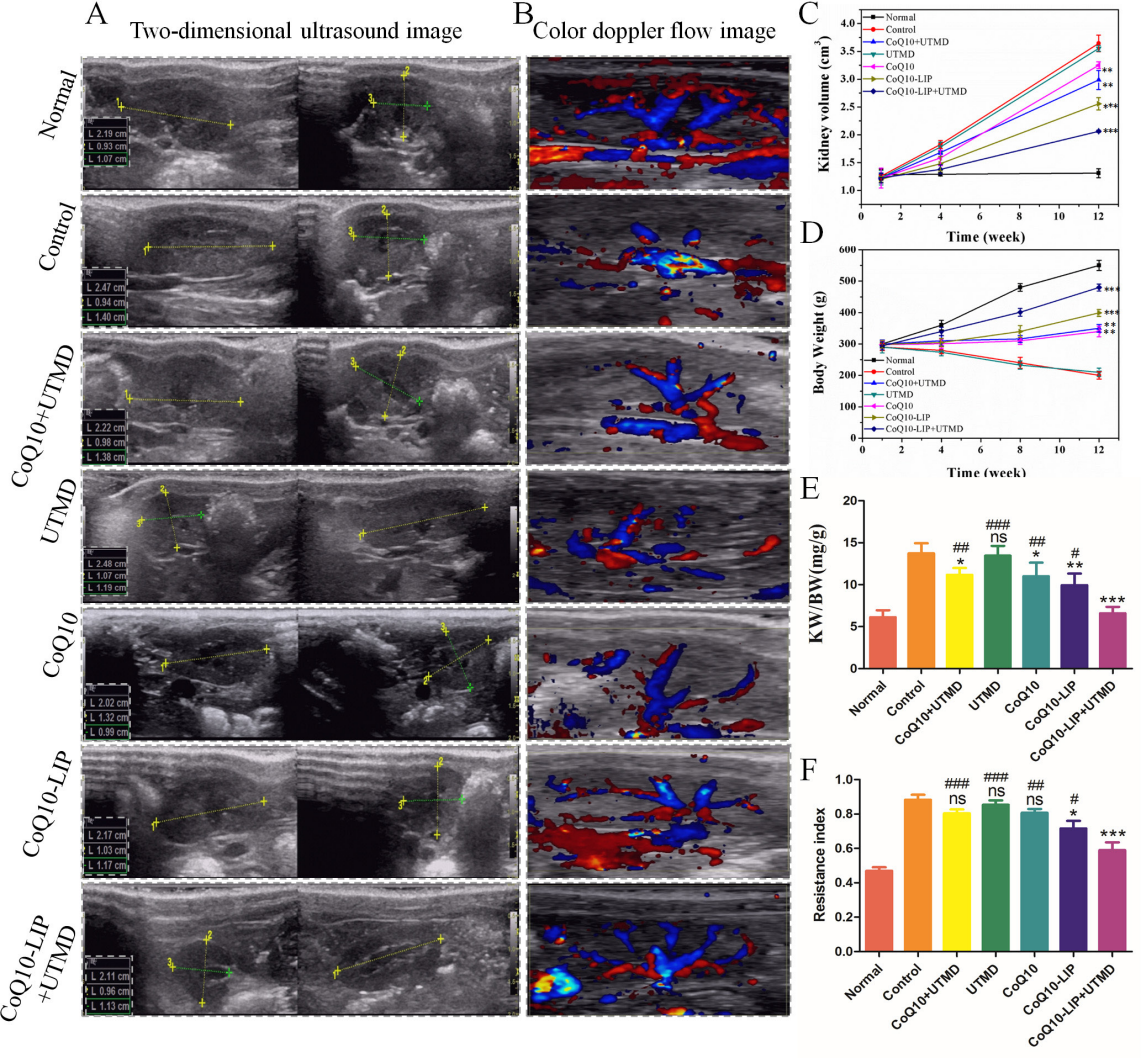
Effects of CoQ10-LIP on kidney morphology and function of diabetic rats (**A**) Two-dimensional ultrasound image of kidney at week 12, (**B**) Color doppler flow image of kidney blood flow at week 12, (**C**) The calculated kidney volume at week 1, 4 and 12, (**D**) the body weight of diabetic rats at week 1, 4, 8 and 12, and (**E**) the calculated KW/BW at week 12, and (**F**) the statistics of resistance index ( ^#^*P* < 0.05, ^##^*P* < 0.01, and ^###^*P* < 0.001 vs. CoQ10-LIP+UTMD group; ^*^P < 0.05, ^**^P < 0.01, and ^***^P < 0.001 vs. control group).

For diabetic rats, long-term existence of hyperglycemia tends to suffer from renal hypertrophy [30]. In order to observe the variation of kidney volume of diabetic rats during treatment with different formulations, two-dimensional ultrasound image were adopted to determine length, width and thickness of the kidney. Results were shown in Figure 2A. The volume of kidney was accordingly calculated by reference formula as mentioned in literature [31]. As shown in Figure 2C, there was no significant difference in kidney volume between diabetic rats and non-diabetic rats at initial one week, indicating that diabetic nephropathy was not obvious at this time. However, after four weeks, the renal hypertrophy was visible, and the kidney volume of diabetic rats was obviously enlarged compared with non-diabetic rats. The kidney volume of the diabetic rats at week 4 and 12 was 1.4-fold and 3.2-fold greater than that of non-diabetic rats. In comparison with the control group, the kidney volume of rats treated with CoQ10 formulations were decreased regardless of imposing UTMD or not. However, CoQ10-LIP treatments exhibited a stronger inhibitory effect on renal hypertrophy of diabetic rats than free CoQ10 treatments. This may due to the longer circulation time in blood of liposomes-encapsulated CoQ10 and more drug distribution in kidney. As expected, CoQ10-LIP+UTMD group showed the most remarkable effect, which may be contributed to the specific distribution of CoQ10-LIP in kidney by UTMD at all tested time point.

On the one hand, with the progress of diabetes, the body weight (BW) of all STZ-treated diabetic rats was gradually declining (Figure 2D). However, DM animals receiving CoQ10, CoQ10+UTMD, CoQ10-LIP and CoQ10-LIP+UTMD showed an increase in body weight during the treatment of 12 weeks. On the other hand, kidney weight (KW) was continuously increasing with the progress of diabetes, and thus KW/BW has been also considered to be an important indicator as progress of diabetic nephropathy. As shown in Figure 2E, KW/BW ratio of control group was significantly higher than normal group as well as the four groups with CoQ10 or CoQ10-LIP with or without UTMD treatment. CoQ10-LIP+UTMD group was significantly lower than other treatment groups. These results suggested the curative effect of CoQ10-LIP+UTMD on diabetic nephropathy was the most strongest among these groups.

Diabetes encompasses a spectrum of vascular complications in the micro-(eye, kidney and nerve) and the macro-vasculature (heart and brain) that are considerably responsible for the recently high morbidity and mortality [32]. Renal microvascular (MV) damage and loss contribute to the progression of renal injury in diabetic nephropathy. The renal blood flow of DM rats after different treatments was also detected by the Color Doppler. The results were shown in Figure 2B. There was little blood flow in the control group. Compared with the control group, the renal blood flow of DM rats treated with CoQ10 solution was improved at some extent. By contrast, the CoQ10-LIP+UTMD group had a richer blood flow in kidney than other CoQ10-treated groups. In addition, renal resistive index (RI) could reflect the changes in intra-renal perfusion/hemodynamics and predict progression of renal disease in hypertensive as well as diabetic nephropathy [33, 34]. We recorded the kidney flow velocity waveform of the RI at least three consecutive cycles by spectral Doppler at week 12. As shown in Figure 2F, RI value in normal group was only about 0.48 ± 0.10, while RI value in control group increased to 0.96 ± 0.13, almost twice over the normal group, indicating poor renal outcome and higher risk for CKD. Compared with the control group, RI values in CoQ10-treatment groups have decreased. Expectedly, RI value in CoQ10-LIP+UTMD group was significantly lower than that of other treatment groups, indicating the better therapeutic effect. In contrast, UTMD alone did not produce any improvement in RI indexes of DM rats, indicating that blood flow was not disrupted by UTMD. Overall, treatment with CoQ10-LIP+UTMD was the most profitable to increase blood flow and may be a promising means to restore blood vessel of damaged kidney.

### Biochemical indictors of rats with diabetic nephropathy after CoQ10-LIP treatment

Long-term existence of high blood glucose usually leads to the occurrence of diabetic nephropathy. Accordingly, the diabetic nephropathy (DN) will be accompanied by the rise of albuminuria, which is considered to be an important indicator of DN [7, 35]. Therefore, blood glucose level and albuminuria content of DM rats with DN were real-time monitored at specific times (1, 4, 8, 12 weeks) during treatment with various formulations.

**Figure 3:**
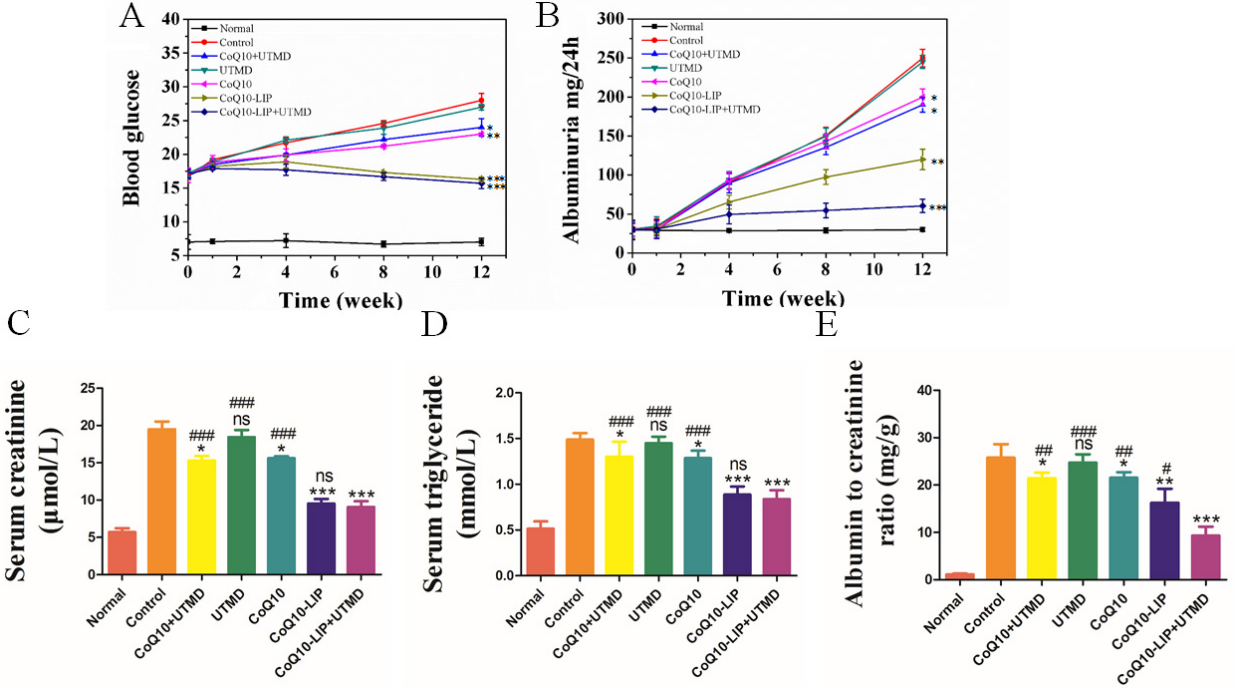
Biochemical indictors of STZ-induced diabetic rats after CoQ10-LIP+UTMD treatment (**A**) Blood glucose, (**B**) Albuminuria, (**C**) Serum creatinine, (**D**) Serum triglyceride, and (**E**) Albumin to creatinine ratio ( ^#^*P* < 0.05, ^##^*P* < 0.01, and ^###^*P* < 0.001 vs. CoQ10-LIP+UTMD group; ^*^P < 0.05, ^**^P < 0.01, and ^***^P < 0.001 vs. control group).

As shown in Figure 3A, DM rats in the control groups had significantly higher blood glucose levels compared with the CoQ10-treated groups at 4, 8, 12 week, indicating the hypoglycemic effect of CoQ10 on DM rats. The similar results were also observed in previous studies [15, 36]. As expected, the hypoglycemic effect after treatment with CoQ10-LIP or CoQ10-LIP+UTMD was more significant than that in DM rats treated with free CoQ10 solution. But there existed no significant difference in hypoglycemic effect between CoQ10-LIP group and CoQ10-LIP+UTMD group. These may due to the drug distribution difference in kidneys between free CoQ10, CoQ10-LIP and CoQ10-LIP+UTMD group. Free CoQ10 was rapidly recognized by reticuloendothelial systems and mainly distributed to liver and spleen because of low solubility in blood. In contrast, CoQ10-LIP may exhibit a long circulation time *in vivo* because of good compatibility with blood components, which made more drugs distributing to kidney. Especially, the specific delivery of CoQ10-LIP to kidneys was easily archived by assistance of UTMD. As an alternative, it was also noted that treatment with CoQ10-LIP+UTMD exhibited an obvious effect on reducing albuminuria level at 4, 8, 12 week (as shown in Figure 3B). Inversely, the albuminuria level in STZ-induced diabetic group was significantly increased in comparison with normal group, indicating appearance of diabetic nephropathy. Besides, both serum creatinine (CRE) and triglyceride (TG) were also considered to be two indicators reflecting the severity of DN for DM rats [4, 6]. Thus, the blood indicators including CRE and TG, which reflected the renal functions, were detected after different treatments. Results were exhibited in Figure 3C–3D. These indicators were remarkably higher in DN rats, while they were significantly decreased in DN rats treated with CoQ10-LIP. Considering that improvement in creatinine and albumin could be a direct effect of reduction in glucose levels, albumin/creatinine ratio was highly associated with renal function. Thus, albumin/creatinine ratio was further plotted in each group. Results were shown in Figure 3E. As expected, albumin/creatinine ratio in DN rats (control group) and UTMD group was remarkably higher than that in healthy rats, while it was decreased in DN rats treated with CoQ10 formulations. Especially, CoQ10-LIP+UTMD treatment presented a lower albumin/creatinine ratio than CoQ10-LIP treatment, suggesting its more effective recovery of the kidney function.

### Anti-oxidative evaluation in DN rats after treatment with CoQ10-LIP+UTMD

**Figure 4:**
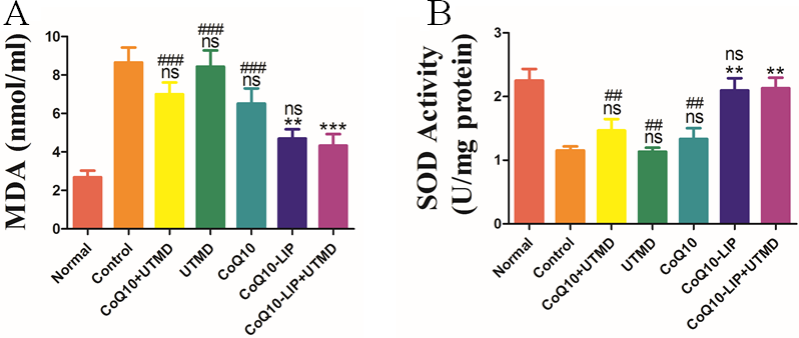
(**A**) Serum malondialdehyde (MDA) and (**B**) serum SOD level in DN rats after various treatments (^#^*P* < 0.05, ^##^*P* < 0.01, and ^###^*P* < 0.001 vs. CoQ10-LIP+UTMD group; ^*^P < 0.05, ^**^P < 0.01, and ^***^P < 0.001 vs. control group).

CoQ10 has a strong antioxidation effect in body, which may also prevent the deterioration of DN in DM rats. *In vivo* antioxidant enzymes such as superoxide dismutase (SOD), catalase (CAT) and glutathione peroxidase (GPx) can metabolize oxidative toxic intermediates in DN rats, and protect normal cells from oxidative stress-induced damage. Alternatively, previous studies have shown that malondialdehyde (MDA) is associated with oxidative stress-induced cell damage in DN rats. Therefore, blood MDA and SOD level in DN rats after different treatments were detected and results were shown in Figure 4A. Serum MDA level in DN rats without treatment was significantly higher than that in normal rats (8.71 nmol/ml vs 2.73 nmol/ml). Serum MDA level in DN rats treated with CoQ10-LIP+UTMD (4.59 mmol/L), CoQ10-LIP (4.66 mmol/L), CoQ10+UTMD (6.96 mmol/L) and CoQ10 (6.26 mmol/L) were notably decreased compare to DN rats without treatment. Inversely, serum SOD level in DN rats was significantly decreased in comparison with normal rats (1.22 U/ml vs. 2.31 U/ml, respectively). DN rats treated with CoQ10-LIP+UTMD (2.09U/ml), CoQ10-LIP (1.98 U/ml), CoQ10+UTMD (1.57 U/ml) and CoQ10 (1.42U/ml) exhibited a higher serum SOD level than DN rats (Figure 4B). These suggested CoQ10 was effective to prevent DN occurrence of diabetic rats due to its anti-oxidative activity. Interestingly, there was no difference in serum SOD level between DN rats treated with CoQ10-LIP and CoQ10-LIP+UTMD. These results showed that UTMD had no significant influence on blood SOD level and MDA level.

### Histopathological staining of kidney after treatment with CoQ10-LIP+UTMD

**Figure 5:**
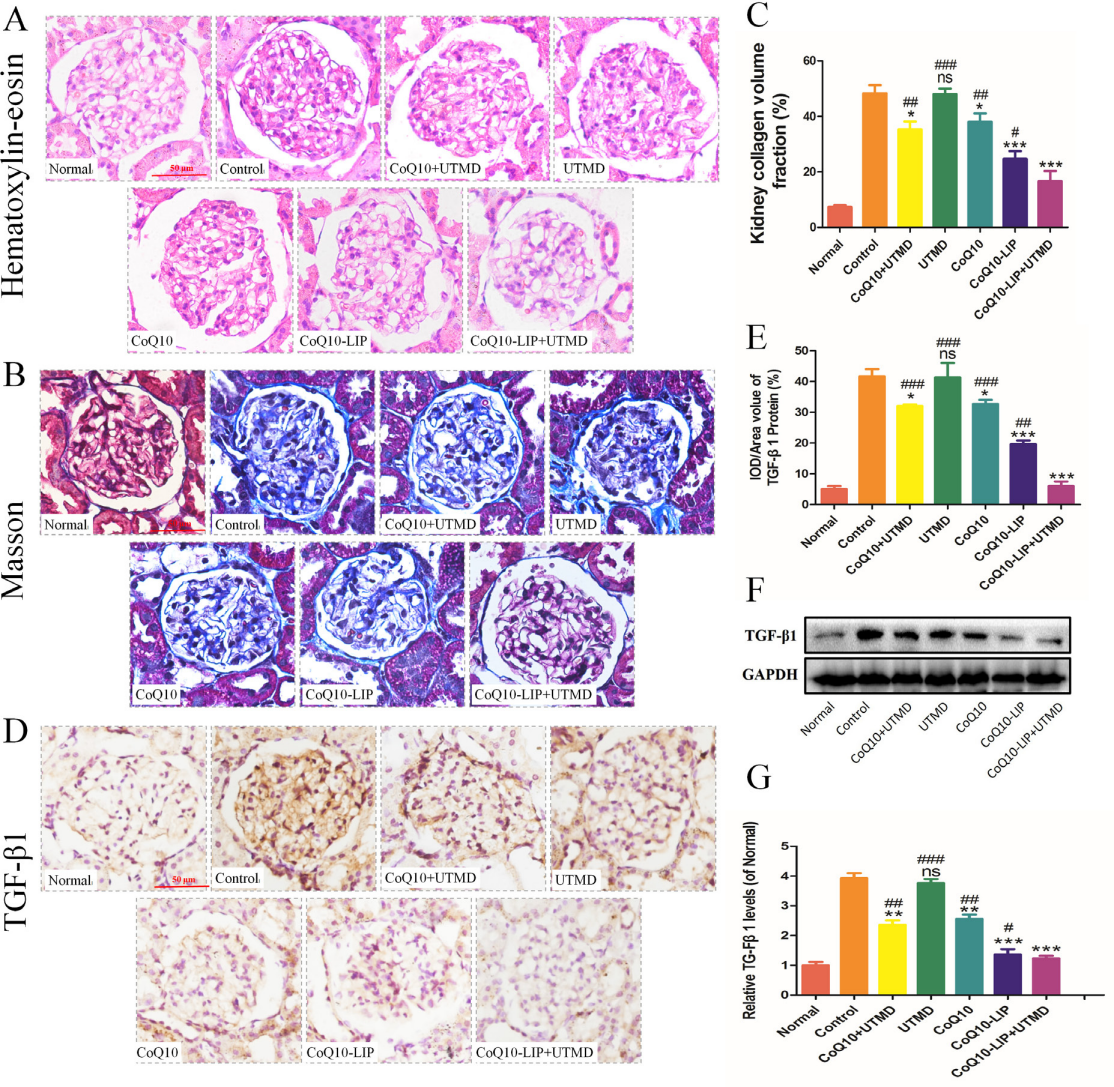
Kidney tissues of DN rats after different treatments was microscopically evaluated (**A**) the histopathological staining, (**B**) Masson staining, (**C**) the quantitative statistics of collagen volume, (**D**) immunohistochemical staining of TGF-β1, (**E**) the quantitative analysis of TGF-β1, (**F**) western blot analysis of TGF-β1 (GAPDH was used as the loading control and for band density normalization) and (**G**) Quantitative analysis of TGF-β1 level. All of the experiments were repeated three times. ( ^#^*P* < 0.05, ^##^*P* < 0.01, and ^###^*P* < 0.001 vs. CoQ10-LIP+UTMD group; ^*^P < 0.05, ^**^P < 0.01, and ^***^P < 0.001 vs. control group).

Morphology of renal tissues in DN rats after various treatments was further evaluated by histopathological staining. As shown in Figure 5A, compared to those normal rats, not only the proliferation of mesangial cell was indicated but also the thickened basement membrane was evidenced in the renal glomerulus of DN rats. Nevertheless, these renal pathological lesions were obviously alleviated in various CoQ10 formulations-treated groups, and the alleviation after CoQ10-LIP+UTMD treatment was the most obvious among these groups. By contrast, treatment with free CoQ10 exhibited only a little therapeutic effect on diabetic nephropathy. This was due to the fact that the water-insolubility of CoQ10 resulted in its low distribution in kidney. Meanwhile, the renal histopathological morphology of DN rats after UTMD treatment was also not significantly improved. Alternatively, from the results of Masson staining in Figure 5B, there were a massive glomerular fibrosis and glomerular cavitation in DN rats in comparison with the normal group. Similarly, glomerular fibrosis and glomerular cavitation were alleviated in various CoQ10 formulations-treated groups. Expectedly, fibrosis in the kidney of DN rats treated with only UTMD was not significantly decreased, while rats treated with CoQ10-LIP+UTMD exhibited a lesser fibrosis than other formulations (Figure 5C). These results were identical with results of HE staining.

Moreover, TGF-β1 was also stained to explore the effect of CoQ10-LIP+UTMD on anti-fibrosis at kidney. Previous study demonstrated that TGF-β1 played an important role in regulating fibrosis, of which overexpression was liable to induce myocardial fibrosis [37, 38]. As shown in Figure 5D, TGF-β1 was highly expressed in kidney of DN rats groups compared with normal group. However, the expression of TGF-β1 was inhibited at some degree after various treatments. TGF-β1-positive cells were quantitatively counted and results were shown in Figure 5E. The trends in expression of TGF-β1 in these groups were identical to that of Masson staining, that was, expression of TGF-β1 decreased by the order: UTMD > free CoQ10≈ free CoQ10+UTMD > CoQ10-LIP > CoQ10-LIP+UTMD. Overall, these results indicated CoQ10-LIP+UTMD treatment reduced the kidney fibrosis through the inhibition of TGF-β1 expression. The similar results were also observed by western blot analysis of TGF-β1 (Figure 5F, 5G).

### Protecting renal podocytes from injury in diabetic rats after CoQ10-LIP+UTMD

**Figure 6:**
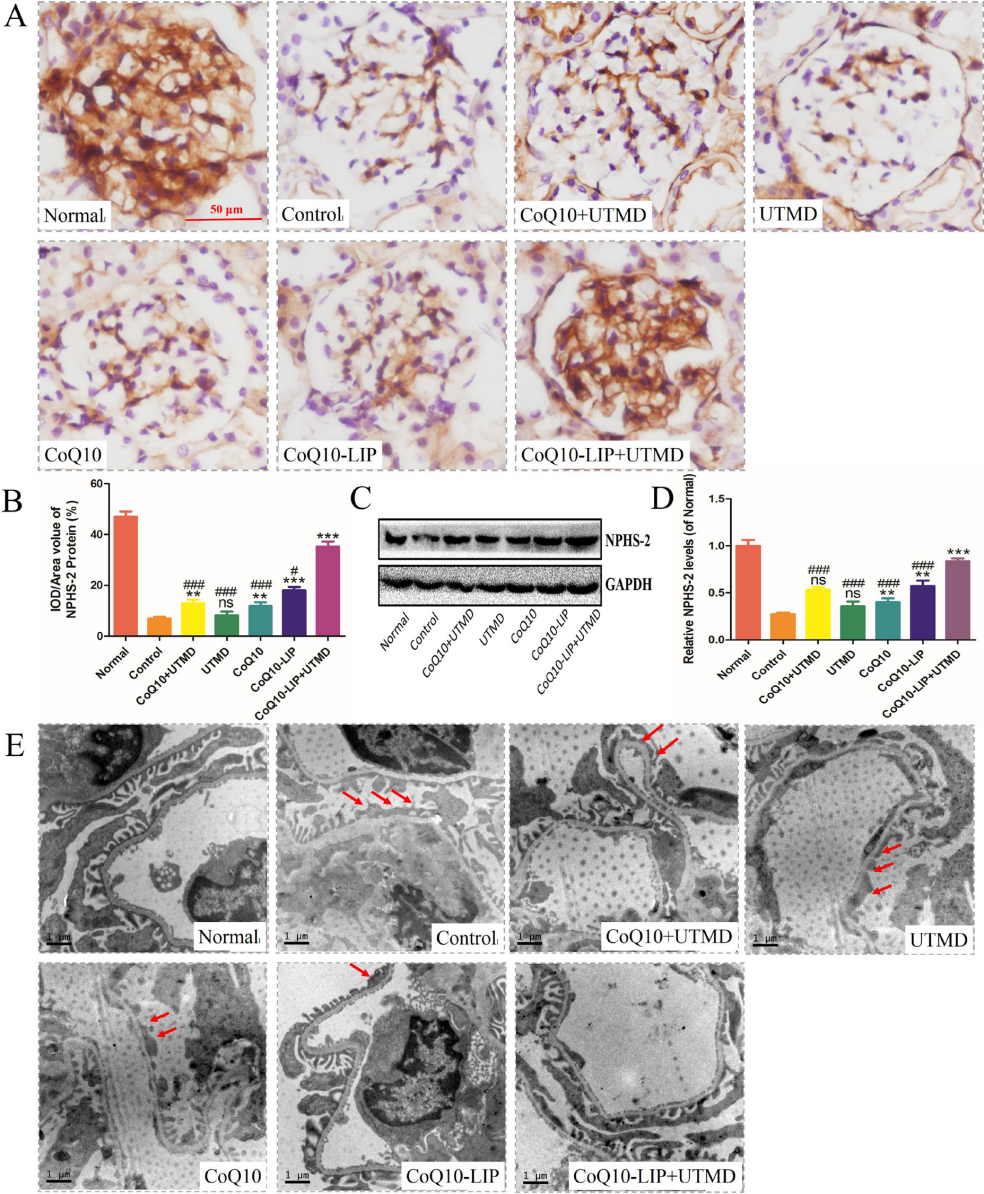
Protecting renal podocyte from injury in DN rats after various treatments (**A**) immunohistochemistrical staining of glomerular NPHS-2, (**B**) The optical density analysis of NPHS-2 protein, (**C**) Western blot analysis of NPHS-2 expression(GAPDH was used as the loading control and for band density normalization), (**D**) Quantitative analyses of the relative NPHS-2 and (**E**) The TEM image in different groups, the red arrow means podocyte fusion. All of the experiments were repeated three times ( ^#^*P* < 0.05, ^##^*P* < 0.01, and ^###^*P* < 0.001 vs. CoQ10-LIP+UTMD group; ^*^P < 0.05, ^**^P < 0.01, and ^***^P < 0.001 vs. control group).

Glomerular podocytes are very pivotal in maintaining the function of glomerular filtration barrier in normal kidney. Severe podocyte damage tends to result in proteinuria in patients with diabetic nephropathy [39]. Podocyte damage, defined as mislocalization of NPHS-2/podocin, a key protein in the slit diaphragm of podocytes, is observed in patients with massive proteinuria, regardless of the underlying disease. NPHS-2 was considered to be a pivotal indicator of podocyte damage. Generally, the decreased expression of NPHS-2 suggested the serious damage of podocyte. As expected, expression of NPHS-2 in healthy rats was broadly localized in glomerular capillary region and mesangial zone (Figure 6A). By contrast, NPHS-2 expression was significantly decreased in DN rats (Figure 6A, 6B), indicating the severe damage of podocyte. The limited recovery of NPHS-2 expression was observed after treatment with free CoQ10 solution or UTMD alone. Although a slight improvement in NPHS-2 expression was restored in these two groups, there was not significantly different from that of control group. However, an obvious improvement of NPHS-2 expression was observed after treatment with CoQ10-LIP+UTMD, indicating the effective protective effect on the damaged podocytes. The similar results were obtained by West blot analysis of NPHS-2 (Figure 6C, 6D). Western blot of NPHS-2 also demonstrated that the CoQ10-LIP+UTMD group had the highest NPHS-2 expression in comparison with other treatment groups. Morphology of podocyte was observed by TEM and results were shown in images (Figure 6E). Compared with normal rats, massive podocyte foot process effacement was revealed in kidneys of DN rats. The podocyte foot process effacement was slightly alleviated in DN rats treated with free CoQ10 solution in comparison with control group. Similarly, the podocyte foot was recovered to the normal morphology after treatment with CoQ10-LIP+UTMD, indicating the beneficial protective effect of CoQ10-LIP on podocytes under assistance of UTMD.

### CoQ10-LIP+UTMD inhibit renal cells apoptosis in diabetic rats

**Figure 7:**
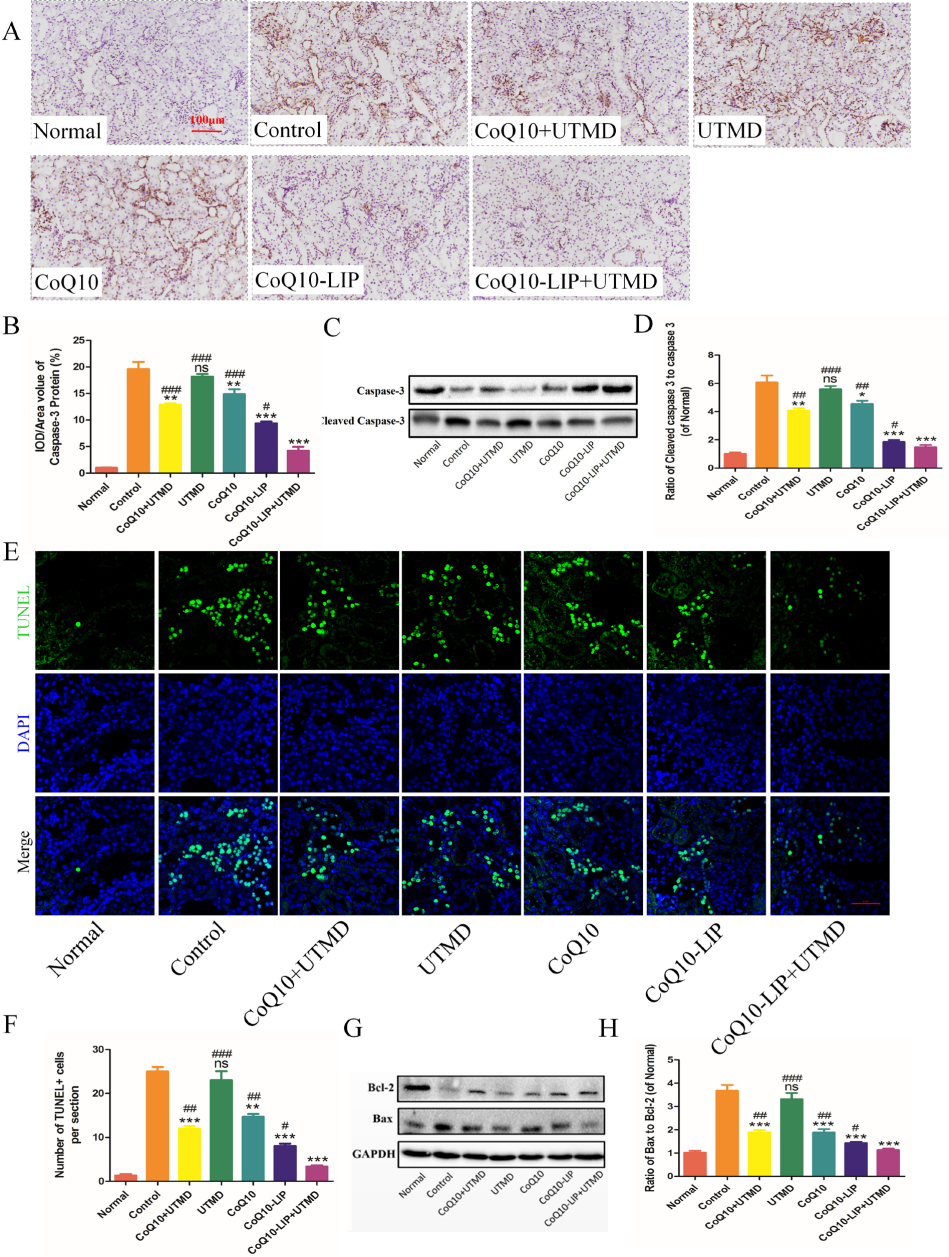
Cells apoptosis of kidney in DN rats after various treatments (**A**) Immunohistochemistrical staining of caspase-3, (**B**) The statistics of renal caspase-3 immunohistochemistry, (**C**) Western blot analysis of renal Cleaved caspase 3 and caspase 3 protein expression, and (**D**) Quantitative analyses of the ratio of Cleaved caspase 3 to caspase 3, (**E**) Immunofluorescent staining of TUNEL (green staining), (**F**) Quantitative analysis of TUNEL-positive cells, (**G**) Western blot analysis of renal Bax and Bcl-2 protein expression, (**H**) Quantitative analyses of Bax/Bcl-2 ( ^#^*P* < 0.05, ^##^*P* < 0.01, and ^###^*P* < 0.001 vs. CoQ10-LIP+UTMD group; ^*^P < 0.05, ^**^P < 0.01, and ^***^P < 0.001 vs. control group).

Severe diabetic renal disease can lead to glomerular atrophy and renal tubular cavity, accompanying by massive cellular apoptosis. Truly, the immunohistochemical staining of renal slice showed that lots of renal cells was going through apoptotic process in DN rats while there was few caspase-3 staining-positive cells in the normal group (Figure 7A, 7B). The cellular apoptosis of renal tissues was not only observed in podocytes of glomerulus but also indicated in renal tubules. The apoptotic phenomenon of renal cells was ameliorated in various CoQ10-formulations treatment groups. In comparison, treatment with CoQ10-LIP+UTMD produced a significant apoptotic inhibition of renal cells. Moreover, among these CoQ10-treated groups, CoQ10-LIP+UTMD group had the lowest apoptosis level. Besides, expression of cleaved caspase 3 and caspase 3 were also detected and the ratio of Cleaved caspase 3/caspase 3 was satisfied. Results were shown in Figure 7C, 7D. Compared to the normal rats, a higher expression of Cleaved caspase 3 was observed while a decreased expression of caspase 3 was seen in the DN group, which presented a higher ratio of Cleaved caspase 3/caspase 3. After treatment with CoQ10-LIP+UTMD, the ratio of Cleaved caspase 3/caspase 3 was significantly decreased (*P* < 0.05), indicating an obvious inhibition of cellular apoptosis.

Alternatively, TUNEL staining of renal slice was also detected and results were shown in Figure 7E, 7F. The similar results were obtained. The apoptotic cellular nuclei were observed in renal slice of control group and UTMD group, but fewer apoptotic cell nuclei were seen in CoQ10-treatment groups. Also, treatment with CoQ10-LIP+UTMD presented the fewest Tunnel staining-positive cells.

Apoptosis-associated molecules including Bax and Bcl-2 were detected by Western blot and results were shown in Figure 7G. Compared to normal rats, expression of apoptotic protein Bax was obviously activated in DN rats, while expression of anti-apoptotic protein Bcl-2 was markedly inhibited. DN rats treated with free CoQ10 solution, exhibited a significantly reduced Bax expression and the up-regulated expression of Bcl-2. Bax/Bcl-2 ratio was directly related with apoptotic level in most cases [40, 41]. Bax/Bcl-2 ratio was further satisfied and results were shown in Figure 7H. Bax/Bcl-2 ratio was markedly increased in DN rats, while the value after treatment with CoQ10-LIP+UTMD was decreased to normal level in normal group.

## DISCUSSION

Accumulating evidences from epidemiological data showed that diabetes considerably increased the risk of renal injury [39, 42]. Currently, for diabetic nephropathy (DN), there is no specific therapy for this condition, which almost invariably progresses to end-stage renal failure [43]. Early prevention of DN in diabetic patients is usually required in clinic. Because of the antioxidant activity, CoQ10 shows a great potential for anti-diabetic and prevention of diabetic nephropathy by strongly scavenging ROS. However, the clinical application of CoQ10 has been limited because of its low aqueous solubility and non-specific distribution. In this study, CoQ10-loaded liposome was prepared to improve the water-solubility and the stability of CoQ10 in circulating blood. Liposomal formulations were usually administrated by local or systemic injection for nephropathy [44]. Local delivery drug allows efficient recovery of injection area, but without diffusion to the whole renal, can’t recover renal function thoroughly. UTMD as non-invasive strategy was a helpful technique to specifically deliver the drug to the whole kidney. The liposomal formulations were usually mixed with microbubbles (MBs) before injection to make them absorbing on the surface of MBs. The mixed liposomal microbubbles were administrated to DN rats through tail vein. Upon exposure to ultrasound localized kidney, the absorbed liposome was pushed through the vessel to renal parenchyma by shear stress produced by MBs under the ultrasound. As shown in Supplementary Figure 1, UTMD can effectively facilitate CoQ10-LIP penetrating the bulk kidney. Before UTMD, microbubbles (MBs) rapidly perfused the vasculature of kidney (the brown dot marked by red arrow). During UTMD, most of MBs were destroyed and facilitated the liposome penetrating deeper tissue of kidney. After UTMD, the fresh MBs re-perfused the vasculature. Besides, more distribution of liposomal drug in kidney under UTMD was further testified by *ex vivo* fluorescence images. FITC-labeled liposome (FITC-LIP) was prepared and replaced CoQ10-LIP in combination with UTMD. The specific distribution of strong fluorescence in left kidney was observed using *ex vivo* fluorescence images after UTMD treatment, while a weak fluorescence was non-specifically distributed in body without UTMD (Supplementary Figure 2A). Moreover, kidney was further sliced and observed by fluorescent microscopy. It was also founded that UTMD effectively enhanced the specific distribution of the absorbed liposome to kidney tissue (Supplementary Figure 2B).

Many studies have shown that CoQ10 exhibit renoprotective effect by blood biochemical markers and histopathological staining. In our study, the blood glucose was observed to decrease in a certain extent after treatment with CoQ10. The similar result was also observed in these publications [15, 36]. Recently, Cristina´s study showed that CoQ10 may play roles in augmenting endogenous antioxidant pathways, decreasing lipid peroxidation and reducing pancreatic lesions of diabetic rats, which would attenuate the loss of glucose homeostasis in these animals. In spite of the hypoglycemic effect of CoQ10, the effect was very limited for DM rats, in which the blood glucose levels were still far higher than the normal level. Long-term existence of high blood glucose was still liable to cause the damage against kidney of diabetic rats [30]. Therefore, controlling the blood glucose may not be the main cause of CoQ10 to prevent DN. In our study, we have further proved the direct protective effect of CoQ10-LIP+UTMD on damaged kidney of DM rats using imaging technology and the detection of the relevant molecules. Firstly, HE and Masson staining revealed that CoQ10-LIP+UTMD exhibited an inhibitory effect on fibrosis of mesangial cells and glomerulus. The molecular mechanism of this effect for CoQ10-LIP+UTMD treatment was highly associated with inhibition of TGF-β1 expression. Secondly, the renal perfusion of blood flow was remarkably improved after CoQ10-LIP+UTMD treatment as indicated by Color Doppler. The nephropathy of diabetic rats has been demonstrated to complicate by microangiopathy with vessels atrophy and necrosis. Protective effect of CoQ10-LIP on microvessel may an important role in preventing the occurrence of DN for DM rats. In addition, damage of glomerular podocytes constituting glomerular filtration barrier tends to produce albuminuria. It was previously reported that exposure of CoQ10 at an appropriate dose can effectively protect the podocyte from damaging and prevent the occurrence of diabetic nephropathy [45]. In our study, the expression of NPHS-2, a marker of active podocyte, was improved in diabetes rats treated with CoQ10-LIP+UTMD. TEM image also obtained the same results. Furthermore, from the result of TUNEL staining, CoQ10-LIP+UTMD treatment group had great effect of inhibiting cellular apoptosis. All above, CoQ10-LIP+UTMD treatment showed the combinational effects including promotion of vascular repair, protection of podocyte, and inhibition of cellular apoptosis in preventing DN occurrence.

Overall, how to maintain its stability in blood circulation and enhance specific distribution of CoQ10 in kidney was very meaningful to improve its therapeutic effect in patients with DN. The CoQ10-LIP combined with UTMD may be a potential strategy to deliver CoQ10 specific for DN precaution.

## MATERIALS AND METHODS

### Materials

Egg Yolk Lecithin (PC, PC-98T) and cholesterol (98% of purity) were purchased from Shanghai Advanced Vehicle Technology Pharmaceutical L.T.D. Co (AVT). Anti-Bax, anti-Bcl-2, anti-NPHS2, anti-caspase-3 and anti-TGFβ1 antibodies were obtained from Abcam (Abcam, Cambridge, MA). Anti-GAPDH primary antibody, and appropriate secondary antibodies were purchased from Santa Cruz Biotechnology (Santa Cruz, CA, USA). CRE, TG and BUN kit were purchased from Nanjing Jiancheng Bioengineering Institute (Nanjing Jiancheng Bioengineering Institute, Nanjing, China). TUNEL kit was obtained from Roche (Roche, Shanghai, China). All other reagents were obtained from Sigma-Aldrich (Sigma-Aldrich, St. Louis, MO). Simulated body fluid (SBF) were purchased from Beijing leagene Biotechnology Company (Beijing leagene Biotechnology Company, Beijing, China). MB was prepared by the sonication-lyophilization method as reported in our recent study [46].

### Preparation and characteristic of CoQ10-liposomes (CoQ10-LIP)

Liposomes containing CoQ10 were prepared employing the thin film hydration method. Briefly, the phospholipid (225mg) and cholesterol (25mg) dissolved in 6 ml dichloromethane to form organic solvent mixture, and 50 mg CoQ10 were dissolved in organic solvent mixture. The organic solvent was then removed by rotary evaporation. The dry lipid film was hydrated with PBS solution. Then, the dispersion was sonicated for 5 min in the ice bath to achieve fully dispersion. To avoid dust and large particles, the emulsion was extruded through a polycarbonate membrane with 0.22 μm pore size.

The encapsulation efficiency of the CoQ10-loaded liposomes was evaluated by ultraviolet-visible spectrophotometer (TU-1901, Puxi, China). The unencapsulated CoQ10 was separated from CoQ10-LIP by the high speed centrifugation at 10,000 g for 40 min, and the supernatant (CoQ10-LIP) was destroyed and properly diluted by DMSO for UV-Vis detection at 275nm. The absorbance was used to calculate the entrapment efficiency according to the standard curve (A = 0.00933C-0.16696, R2 = 0.99952). Finally, the encapsulation efficiency was calculated by the formula as follow: Encapsulation efficiency (%) = (Total CoQ10 amount – amount of free CoQ10) / Total amount of CoQ10 × 100%.

The particle sizes and size distribution of CoQ10-LIP was measured by dynamic light scattering detector (Zetasizer Nano ZS90, Malvern, UK) at 25°C. The zeta potential was determined using a Zeta PALS (American PSS Ltd., America). Morphology of CoQ10-LIP was observed by using transmission electron microscope (TEM, JEM-100CX, Japan). A drop of CoQ10-LIP dispersions was placed and adsorbed on microscopic carbon-coated copper grids. These grids were subsequently stained with 1% (w/v) aqueous solution of phosphotungstic acid, dried, and viewed under TEM at suitable magnifications, operating at an acceleration voltage of 200 kV.

### *In vitro* release of CoQ10 from CoQ10-LIP

*In vitro* release of CoQ10 from liposomes was determined according to the method as previously described. CoQ10 liposomes suspension (2 ml) was placed into dialysis bag (3500 Da), and then incubated with 50 ml of release medium (Tween 80: Ethanol: PBS buffer = 5:20:75, v/v/v) under continuous stirring (120 rpm/min) at 37 °C. At each time interval, 1 ml of release medium was withdrawn and drug concentration was determined by UV-vis spectrophotometry at 275 nm according to the standard curve. At same time, equivalent volume of fresh medium was supplemented at each sample time. The cumulative release percentage was calculated by the formula as follow: The cumulative release percentage (%) = (amount of the released CoQ10 / total CoQ10 amount in liposome) × 100%.

### Animal treatment

### Type 1 Diabetic rat model

This study was reviewed and approved by the Ethics Committee for Experimental Animals of Wenzhou Medical University. 56 male Sprague-Dawley rats (150–200 g) were purchased from Shanghai, China. Type 1 DM animal model was established according to the reported method [46]. In brief, SD rats was received intraperitoneal single injection of streptozotocin (STZ) at 70 mg/kg after 12 h of fasting. STZ (Sigma-Aldrich, St Louis, MO, USA) was prepared as 1% STZ solution in 0.1 M citrate buffer (pH 4.0–4.5). On the 3rd day, 7th day, and 2nd week after STZ administration, the fasting blood glucose was measured from the tail tip using an autoanalyzer (Surestep, Roche, Germany). Only the animals with a fasting blood glucose exceeding 16.7 mM after STZ treatments were used as the study animals.

### Combinational treatments of CoQ10-LIP and UTMD

The experimental rats were randomized into seven groups: (1) Normal group: non-diabetic rats were administered normal saline; (2) Control group: DM rats were administered normal saline; (3) CoQ10 group: DM rats were administered CoQ10 in normal vehicle; (4) UTMD group: DM rats were treated with US; (5) CoQ10+UTMD group: DM rats were treated with CoQ10 solution with US; (6) CoQ10-LIP group: DM rats were treated with CoQ10-LIP solution without US; (7) CoQ10-LIP+UTMD group: DM rats were treated with CoQ10-LIP solution with US. The dose of CoQ10 in CoQ10-LIP or CoQ10 solution was 15mg/kg weight. For all animals, every reagent were administered via tail vein injection twice weekly for first four weeks to achieve a preventive effect of DN.

The experimental animals were anesthetized with intraperitoneal injection of 30 mg/kg sodium pentobarbital. The animals were placed in the prone position, and the left abdomen was shaved. A linear array transducer was placed over the left kidney (short axis view; depth = 1.0–2.0 cm) to view the left kidney. The drugs were injected through the tail vein followed by the UTMD treatment. A linear array transducer (9L probe, 5–9 MHz, Logic E9, GE Medical system, USA) was used to generate the UTMD effect. When a large number of microbubbles appeared in the kidney, the microbubble destruction (MBD) function key attached to the machine was used to blast the microbubbles for the UTMD (mechanical index [MI] = 1.3, exposure time = 10 seconds , repeat 6 times with off intervals of 1s). The probe was moved slowly (up and down or side to side) to scan entire left kidney, until the microbubbles completely disappeared in the left kidney. During this process, the impact force of MBs will push the CoQ10-LIP through the blood vessels and penetrate the entire renal parenchyma.

### Effects of CoQ10-LIP+UTMD on morphology and function of kidney of diabetics rats

### Ultrosound-gulided imaging of kidneys

Ultrasound imaging is particularly attractive technique to detect the morphology and blood flowing of soft tissues owing to the portability of imaging machines and the inherent safety of low-power acoustic interrogation of tissue [24]. Kidneys were imaged by two-dimensional ultrasound technique and the parameters including the length, width and thickness of kidney were probed. The renal volume was accordingly calculated by the following formula: Kidney volume (cm^3^) = (π/6) × length (cm) × width (cm) × thickness (cm). A color Doppler model was used to observe the blood flow distribution in the left kidney of the rats. The waist hair of rats was shaved, and placed in the prone position. A linear array transducer (ML6-15 probe, 15 MHz, General Electric Company, USA) was used for color Doppler. The probe was then rocked gently up and down or side to side to scan the entire kidney. Then the spectral Doppler mode was used to collect hemodynamic parameters such as the resistance index (RI).

### Physical and biochemical analysis

Body weight, blood glucose and albuminuria level were measured every 4 weeks. At 12th week, the animals were sacrificed, the blood was collected immediately and serum levels of creatinine (CRE), triglyceride (TG) was measured by the CRE/TG assay kit (Nanjing Jiancheng Bioengineering Institute, Nanjing, China). The kidney weight was measured and the KW/BW ratio was calculated (KW/BW = Kidney weight (mg) / Body weight (g)). In addition, Superoxide dismutase (SOD) activity and malondialdehyde (MDA) level in serum were measured using the SOD/MDA assay kit (Nanjing Jiancheng Bioengineering Institute, Nanjing, China) according to the manufacturer’s instructions. 24-h urinary protein from all rats were monitored at specific times (0, 1, 4, 8, and 12 weeks), the urinary albumin concentration was assayed using the urine protein kit (Nanjing Jiancheng Bioengineering Institute, Nanjing, China) according to the manufacturer’s instructions.

### Renal histopathological and molecular biology analysis

### HE and Masson stain

Tissues were fixed in 4% paraformaldehyde and subsequently embedded in paraffin. Sections (5 μm thick) were stained with hematoxylin and eosin (HE) using a standard protocol and analyzed by optical microscopy (Nikon ECLPSE 80i, Japan) to observed the mesangial cell proliferative. The collagen content of the kidney was detected by Masson’s trichrome kit (Sigma-Aldrich, USA). All above assay were experiment as previous.

### Immunohistochemistry in rats renal tissue

Paraffin-embedded sections were stained for caspase-3 and NPHS-2. First, the sections were deparaffinized and rehydrated and then immersed in 3% H_2_O_2_ for 15 min at 37°C to block the endogenous peroxidase activity. Then the antigen retrieval were adopt by EDTA for 30 min at 37°C. After that, the samples were blocked using 5% bovine serum albumin (BSA) (Beyotime, China) for 30 min at 37°C. Subsequently, primary antibodies diluted in PBS containing 1% BSA were used rabbit polyclonal anti-caspase-3 and rabbit polyclonal anti-NPHS-2 for 2h at 37°C, HRP-secondary antibody for 1 h at 37°C and stained with DAB and counterstained with hematoxylin. The stained sections of kidney were examined and recorded with an optical microscope.

### TUNEL assay

Apoptotic cell in kidney sections was detected by using the TUNEL kit (Roche Germany). All the processes were performed according to the manufacturer’s instructions. Paraffin sections were dewaxed, treated with H_2_O_2_ and incubated with the reaction mixture containing terminal deoxynucleotidyl transferase (TdT) and digoxigenin-conjugated dUTP for 1 h at 37°C. After TUNEL labeling, nucleus was labeled with DAPI and the TUNEL positive labeled apoptotic cells were observed using a Nikon confocal laser microscope (Nikon, A1 PLUS, Tokyo, Japan).

### Transmission electron microscopy (TEM) image

After fixation in 2.5% (w/v) glutaraldehyde overnight, tissue were post-fixed in 2% (v/v) osmium tetroxide and blocked with 2% (v/v) uranyl acetate. Following dehydration in a series of acetone washes, tissue were embedded in Araldite. Semi-thin section and toluidine blue staining were performed to observe the location. Finally, ultra-thin sections of at least three blocks per sample were cut and observed using a Hitachi TEM.

### Molecular analysis

Rats renal were homogenized in buffer supplemented with protease inhibitors. Samples with equal amounts of total protein (50 mg/ml) were mixed in Laemmli loading buffer, boiled for 10 min, and then processed for Western blotting as described previously. Briefly, 20 μl of each samples was separated on sodium dodecyl sulfate polyacrylamide gel, after electrophoresis, proteins were transferred onto PVDF membrane (Millipore Company, USA) and blocking with 5% (w/v) non-fat milk for 2 hours, then blotted against primary antibody (Bax, Bcl-2, NPHS-2, GAPDH) over night at 4°C. Next day, the membranes were incubated with a secondary antibody for 2 hours at room temperature. Finally, signals were visualized using the ChemiDicTM XRS + Imaging System (Bio-Rad).

### Statistical analysis

Statistical analysis was carried out using the statistical software program Statistical Package for Social Sciences (SPSS). Data were expressed as the means ± SD. All the results were statistically analyzed by the unpaired Student’s *t*-test and multiple comparisons were carried out using one-way ANOVA followed by post hoc analysis [47, 48]. *P* < 0.05 was considered statistically significant.

## SUPPLEMENTARY MATERIALS FIGURES AND TABLES


