# Supplementary material for: CoQ10-loaded liposomes combined with UTMD prevented early nephropathy of diabetic rats
Source: Oncotarget. 2018 Jan 19;9(14):11767–82. doi: 10.18632/oncotarget.24363 (PMC5837748; doi:10.18632/oncotarget.24363)
Supplement: Supplementary file 1 [file oncotarget-09-11767-s001.pdf]

CoQ10-loaded liposomes combined with UTMD prevented early nephropathy of diabetic rats

SUPPLEMENTARY MATERIALS

Distribution of CoQ10-LIP in whole body under UTMD

In order to investigate CoQ10 delivery or uptake into kidney, the FITC labeled liposome was prepared by the similar method as CoQ10-LIP. In brief, 100 mg of total lipids (PC) and cholesterol with molar ratio (10:1) were dissolved in 10 ml of dichloromethane as oil phase. 1mg of FITC was added to the oil phase. After sonication, oil phase was further evaporated on a rotary evaporator to remove organic solvent under vacuum. After that, the residual dry lipids membrane was hydrated with ph7.4 PBS (10 mM) at room temperature. Finally, the mixed FITC-LIP was extruded through a polycarbonate membrane with 100 nm pore size for 10 times.

After that, 0.1 ml of FITC-LIP suspension was mixed with PMBs suspension and the mixed suspension was administered via intravenous injection. And then, ultrasonic process was imposed on left kidney (MI = 1.3, exposure time = 10 s, repeat six times with off intervals of 1 s to allow refill of the tissue with more microbubbles). After ultrasound, the in vivo fluorescence images of whole body were instantly observed by using an In Vivo IVIS spectrum imaging system (wavelength of 500 nm at an excitation wavelength of 485 nm). The main organs including heart, liver, spleen, lung and kidney was collected and sliced for fluorescent imaging.

RESULTS

In vivo fluorescence images were shown in Supplementary Figure 2A. The specific distribution of strong fluorescence in left kidney was observed using ex vivo fluorescence images after UTMD treatment, while a weak fluorescence was non-specifically distributed in body without UTMD. Moreover, the fluorescent distribution of CoQ10-LIP in main organs was shown in Supplementary Figure 2B. FITC-LIP was mainly distributed to liver, spleen and lung of DN rats without UTMD and few of them was delivered to left kidney. By contrast, the fluorescent distribution in non-targeted organs such as liver, spleen and lung was significantly decreased, while more FITC-LIP was specifically delivered to kidney under the assistance of UTMD. These suggested that UTMD effectively enhanced the specific distribution of the absorbed liposome to kidney tissue.

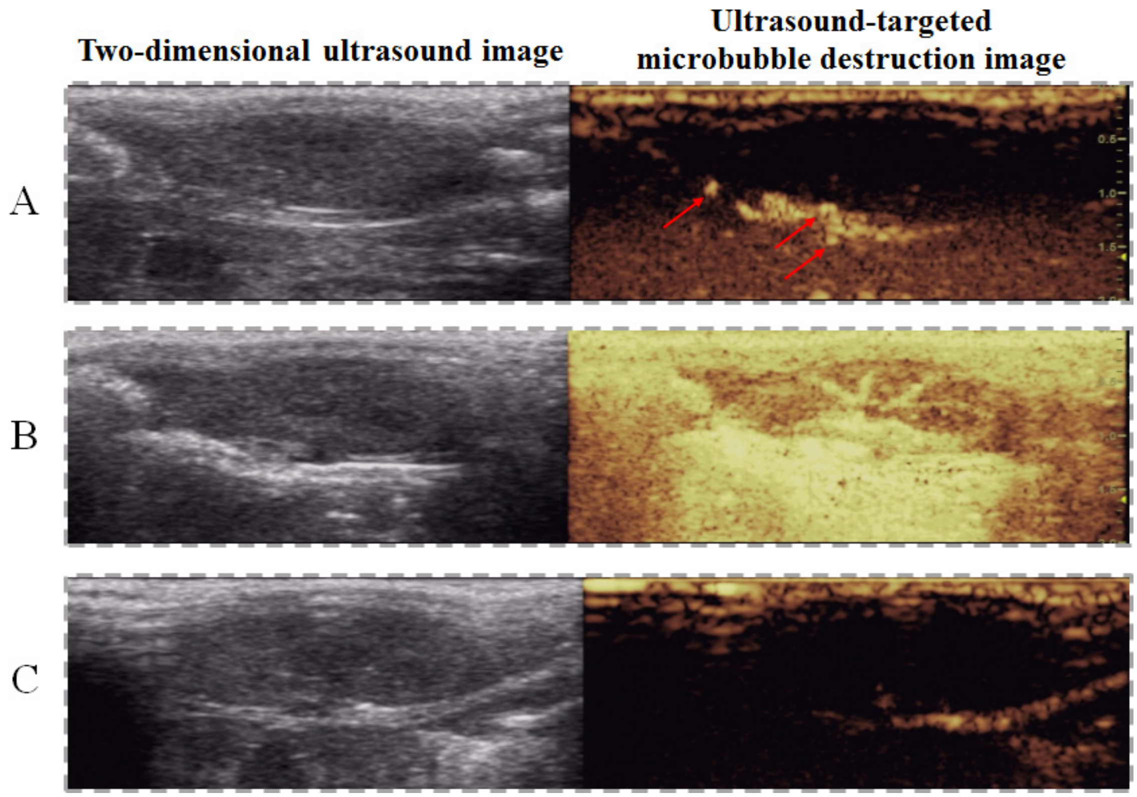

Supplementary Figure 1: The process of ultrasound targeted microbubble destruction. (**A**) Before UTMD, microbubbles (MBs) rapidly perfused the vasculature of kidney (the red arrow represent CoQ10-LIP carried by MB), (**B**) During UTMD, most of MBs were destroyed and facilitated the liposome penetrating deeper tissue of kidney, and (**C**) After UTMD, the fresh MBs re-perfused the vasculature.

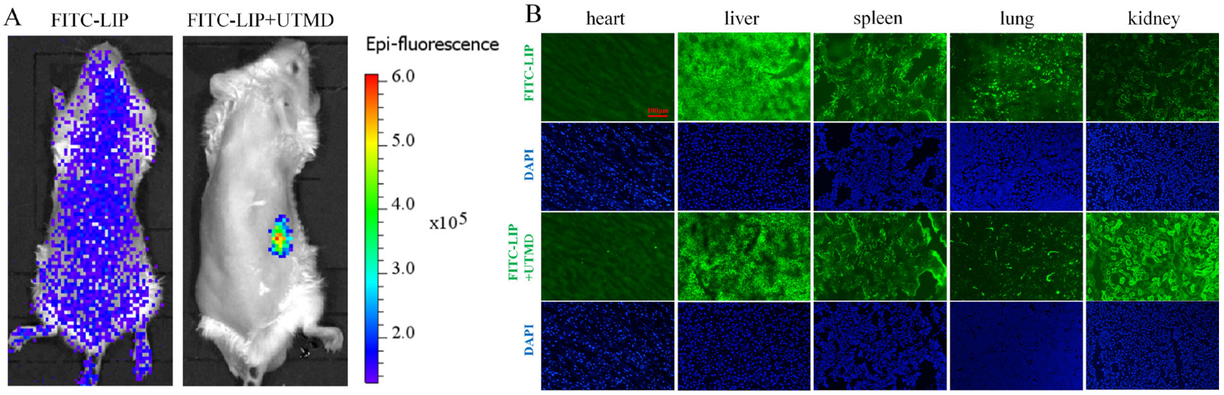

Supplementary Figure 2: Distribution of FITC-LIP under UTMD in whole body. (**A**) Ex vivo FITC fluorescence images of FITC-LIP and FITC-LIP+UTMD. (**B**) Distribution of FITC-LIP in heart, liver, spleen, lung, kidney with or without UTMD.
